# Effect of Melatonin in Epithelial Mesenchymal Transition Markers and Invasive Properties of Breast Cancer Stem Cells of Canine and Human Cell Lines

**DOI:** 10.1371/journal.pone.0150407

**Published:** 2016-03-02

**Authors:** Naiane do Nascimento Gonçalves, Jucimara Colombo, Juliana Ramos Lopes, Gabriela Bottaro Gelaleti, Marina Gobbe Moschetta, Nathália Martins Sonehara, Eva Hellmén, Caroline de Freitas Zanon, Sônia Maria Oliani, Debora Aparecida Pires de Campos Zuccari

**Affiliations:** 1 Department of Molecular Biology, Faculdade de Medicina de São José do Rio Preto, São José do Rio Preto, SP, Brazil; 2 Department of Biology, Universidade Estadual Paulista “Júlio de Mesquita Filho”, São José do Rio Preto, SP, Brazil; 3 Department of Anatomy, Physiology and Biochemistry, Swedish University of Agricultural Sciences, Uppsala, Sweden; University of Toronto, CANADA

## Abstract

Cancer stem cells (CSCs) have been associated with metastasis and therapeutic resistance and can be generated via epithelial mesenchymal transition (EMT). Some studies suggest that the hormone melatonin acts in CSCs and may participate in the inhibition of the EMT. The objectives of this study were to evaluate the formation of mammospheres from the canine and human breast cancer cell lines, CMT-U229 and MCF-7, and the effects of melatonin treatment on the modulation of stem cell and EMT molecular markers: OCT4, E-cadherin, N-cadherin and vimentin, as well as on cell viability and invasiveness of the cells from mammospheres. The CMT-U229 and MCF-7 cell lines were subjected to three-dimensional culture in special medium for stem cells. The phenotype of mammospheres was first evaluated by flow cytometry (CD44^+^/CD24^low/-^ marking). Cell viability was measured by MTT colorimetric assay and the expression of the proteins OCT4, E-cadherin, N-cadherin and vimentin was evaluated by immunofluorescence and quantified by optical densitometry. The analysis of cell migration and invasion was performed in Boyden Chamber. Flow cytometry proved the stem cell phenotype with CD44^+^/CD24^low/-^ positive marking for both cell lines. Cell viability of CMT-U229 and MCF-7 cells was reduced after treatment with 1mM melatonin for 24 h (*P*<0.05). Immunofluorescence staining showed increased E-cadherin expression (*P*<0.05) and decreased expression of OCT4, N-cadherin and vimentin (*P*<0.05) in both cell lines after treatment with 1 mM melatonin for 24 hours. Moreover, treatment with melatonin was able to reduce cell migration and invasion in both cell lines when compared to control group (*P*<0.05). Our results demonstrate that melatonin shows an inhibitory role in the viability and invasiveness of breast cancer mammospheres as well as in modulating the expression of proteins related to EMT in breast CSCs, suggesting its potential anti-metastatic role in canine and human breast cancer cell lines.

## Introduction

Breast cancer is the most prevalent cancer in women worldwide representing 23% of all cases of cancer [1]. Mammary tumors are also common in female dogs, representing approximately 52% of all neoplasms that affects this animal population [2, 3]. In both species, breast cancer has a high rate of mortality and morbidity mainly due to the tumoral recurrence and metastasis [4].

Cancer stem cells (CSCs) are responsible for tumor initiation, recurrence, metastasis and resistance to therapy in several tumor types, including breast cancer [5–7]. These cells, also called tumor-initiating cells, constitute a distinct fraction in the tumor mass, and they have the capacity of self-renewal and pluripotency, reproducing the heterogeneity of the original tumor from which they are derived [6, 8]. These features also characterize embryonic stem cells, therefore suggesting common molecules might exist between CSCs and embryonic stem cells, such as, octamer-binding transcription factor 4 (OCT4), a essential regulator for the self-renewal and pluripotency [6].

The subpopulation of breast CSCs is characterized by phenotype CD44^+^/CD24^low/-^ [9, 10], by their tumor-initiation capability in immune-compromised mice [11] and by an *in vitro* ability to form mammospheres [12, 13]. The undifferentiated cells derived from epithelium are the only ones capable to survive in suspension and to form mammospheres, the other cell types die by anoikis [12].

The epithelial mesenchymal transition (EMT) is a mechanism to generate cancer stem cells endowed with an invasive and metastatic phenotype [14, 15]. EMT occurs in the embryogenesis process, during the organ and tissue formation as well as in trauma restoration and organ fibrosis and carcinogenesis [16, 17]. This process is mediated by the activity of growth and transcription factors, resulting in loss of the epithelial cells’ typical intercellular junction structure, acquisition of mesenchymal morphology, loss of apical-basal cell polarity and motility and invasion ability [18]. Studies have also demonstrated that EMT is involved in cell plasticity, process by which non-stem cells acquire stem cell characteristics [19].

The major EMT molecular marking include loss of the epithelial marker E-cadherin, and overexpression of mesenchymal markers as N-cadherin and vimentin [16]. E-cadherin, a member of the cadherin superfamily, is a structural component of adherent junctions, fundamental to the polarity and adhesion of epithelial cells [18, 20, 21]. N-cadherin, another member of the cadherin family responsible for the integrity of adherent junctions, is usually expressed in mesenchymal cells [22, 23]. Vimentin, is a main component of the intermediate filament family of proteins and it is expressed in the mesenchymal cells [24].

Currently there has been growing interest in identifying new therapeutic agents that may interact with molecular markers present in cancer stem cells, formed in the EMT process. Thus, these new agents could interfere in the metastatic process, which is the main cause of mortality among cancers, including breast cancer [5].

Melatonin (N-acetyl-5-methoxytryptamine), a naturally hormone produced and secreted by pineal gland, has been proven effective in tumor inhibition, in both *in vitro* and *in vivo* studies [25–27]. This hormone has oncostatic activity through a variety of mechanisms including antiproliferative actions, modulation of oncogenes expression, antioxidant and antiangiogenic effects [28].

According to Lopes et al. [29], melatonin inhibits cell viability and proliferation and induces apoptosis in canine breast cancer cells, especially ER-positive with high expression of MT1 receptor. Studies also suggest that melatonin has anti-invasive and anti-metastatic action, which involves multiple cellular models including EMT [30–32]. According to Mao et al. [33], melatonin has inhibited EMT in MCF-7 cells because it induces the degradation of β-catenin, an E-cadherin repressor, via activation of kinase protein GSK3β. Nowadays some studies have shown inhibitory effect of melatonin in cancer stem cells. Thus, previous studies demonstrated that the treatment with melatonin was able to decrease the cell proliferation and induced the cell death by apoptosis and autophagy of colorectal and glioma CSCs [34, 35].

However, the specific action of melatonin in cancer stem cells, which result from EMT, has been underexplored [30, 36]. Therefore, the objectives of this study were to evaluate the effects of melatonin treatment on modulation of molecular markers: OCT4, E-cadherin, N-cadherin and vimentin, as well as, in the cell viability and invasiveness of the cell mammospheres.

## Materials and Methods

### Cell cultures

The canine mammary cancer cell line CMT-U229, previously cultivated [37] was kindly provided by Dr. Eva Hellmén. The histological type this canine cell lineage it is a benign mixed mammary tumor according to WHO (World Health Organization). The human breast cancer cell line MCF-7 was obtained from ATCC (American Type Culture Collection, Manassas, VA, USA).

The CMT-U229 and MCF-7cells were grown in a humidified incubator at 5.0% CO_2_ at 37°C until they were 80–90% confluent in Dulbecco’s modified Eagle’s medium (DMEM) high glucose (Cultilab, Campinas, SP, Brazil) and HAM-F12 (Cultilab, Campinas, SP, Brazil), respectively, supplemented with 10% fetal bovine serum (FBS) (Cultilab, Campinas, SP, Brazil), penicillin (100 IU/mL) and streptomycin (100 mg/mL) (Sigma-Aldrich, St. Louis, MO, USA).

### Mammospheres culture assay

Mammosphere culture was performed as previously described [38]. Cells were grown in the MammoCult medium (Stem Cell Technologies, Vancouver, Canada) supplemented with MammoCult Proliferation Supplements (Stem Cell Technologies, Vancouver, Canada) and plated in ultra low attachment individual wells of 8.96 cm^2^ at a density of 10,000 viable cells/mL and grown for 7 days. The mammosphere formation rate was calculated as previously described [39] using the following equation: (number of mammospheres per well/number of cells seeded per well) × 100%.

### Flow cytometry analysis

By using a Guava easyCyte flow cytometer (Millipore), the expression of stem cell markers in a breast cancer panel was distinctly evaluated in cells from mammospheres. The antibodies used were phycoerythrin (PE)-conjugated anti-CD24 (BDbiosciences) and fluorescein isothiocyanate (FITC)-conjugated anti-CD44 (BDbiosciences). Staining was done according to the instructions of the manufacturer and analysed in flow cytometer. For the analysis, 80,000 cells were detected and quantified by percentage (%).

### MTT cell viability assay

The cell viability potential of cells treated or not with 1 mM melatonin (Sigma-Aldrich, St. Louis, MO, USA) was measured by MTT assay (3(4,5- dimetiliazol-2-il)-2,5difeniltetrazolium bromide), which is based on the ability of live cells to convert tetrazolium salt into purple formazan.

To MTT assay, the mammospheres were disaggregated by the action of the enzyme trypsin and the cells were plated in a 96 well plate. Thus, the MTT assay was performed with adherent cells provided from mammospheres.

Regarding treatment, melatonin was diluted with 50% of PBS (Phosphate Buffered Saline) solution and 50% of ethanol and the cells received treatment in the absence of light, because the hormone is photosensitive. Melatonin has been shown to act decreasing the cell viability of breast cancer cell lines [40, 41]. It should be emphasized here that the concentration of 1 mM melatonin used for the treatment of the cells was defined according to the literature. This is the pharmacological concentration used in several studies about the effects of melatonin in neoplastic cells [42–47].

Briefly, individual well of 0.31 cm^2^ was inoculated with 100 μL of supplemented medium containing 5 x 10^4^ cells. After 24 h, 10 μL MTT stock solution (5 mg/mL, Sigma Chemical Co) was added to each well, and the plates were further incubated for 4 h at 37°C. To solubilize the MTT formazan crystals, the cells were incubated with SDS-HCl (10 mM) (Invitrogen Life Technologies, Carlsbad, CA, USA) at 37°C for 4 h. The absorbance at a wavelength of 570 nm was measured by the ELISA reader (Thermo Plate, Waltham, MA,USA). For the analysis, the cell viability (%) was calculated for all groups compared to control samples. All experimental samples were done in triplicate.

### Immunofluorescence staining

Cells from mammospheres, attached to 8-well chamber slides (Sarstedt, Newton, NC, USA) and incubated or not with 1 mM melatonin at 37°C for 24 h, were fixed immediately in 4% paraformaldehyde and permeabilized 0.4% Triton X-100 for 20 minutes. Cells were blocked with 10% normal goat serum (Sigma-Aldrich, St. Louis, MO, USA) and then incubated with the antibodies E-cadherin (1:400 Abcam), N-cadherin (1:400 Abcam), Vimentin (1:100 Sigma Aldrich, St. Louis, MO, USA) and OCT-4 (1:1000 Abcam) at 4°C overnight, followed by incubation with secondary Alexa Fluor 488 anti-rabbit IgG (Sigma-Aldrich, St. Louis, MO, USA) per 1 hour at room temperature. Nuclear staining was performed by 4',6-diamidino-2-phenylindole (DAPI, Life Technologies, Eugene, OR, USA) and mounted with Prolong Gold (Life Technologies, Eugene, OR, USA). Images were captured on a confocal microscopy (ZEISS, modelo LSM 710, software ZEN 2010, Thornwood, NY, USA). The spheroids were measured at 405 nm for nuclei staining and 488 nm for cytoplasmic staining.

### Immunofluorescence staining evaluation

Two different photomicrographs were taken at 40X magnification under bright field and the intensity of the staining was quantified by Image J Software (NIH, Bethesda, MD, USA). Each photograph was divided into four quadrants and 20 spots (small circular ROI) were randomly selected in each photomicrography. A negative control section of the corresponding staining was used to measure background activity in S1 Fig. The slides were observed in a microscope Nikon Eclipse E200. The values were obtained in arbitrary units (a.u.) and showed the mean optical density (M.O.D.) for each sample. The pinhole size was 90 μm.

### Invasion Assay

The invasion assay was performed with the adherent cells provided from mammospheres.

The invasiveness of CMT-U229 and MCF-7 cells treated or not with melatonin was assessed using transwell chambers with 8 μM pore size membrane (Corning Matrigel Invasion Chamber; Bedford, MA, USA). In the upper compartment of the chamber, approximately 2.5 x 10^4^ cells/insert were added into the culture medium without serum, while to the other compartment were added 750 μL of culture medium with the chemoattractant (0.5% and 10% FBS to negative and positive control’s, respectively) and 10% FBS was associated with 1 mM melatonin.

The cells were incubated at 37°C in 5% of CO_2_ for 24 h. After incubation, the transwell membranes were washed and impermeabilized. The invading cells were stained with hematoxylin and the nonmigrating cells were removed from the upper surface of the transwell membrane by a cotton swab. The counting was made with an inverted optic microscope by putting the insert over a plate containing glycerol at 50%.

Cell invasion (%) of each group was calculated from the average values of cells that migrated and invaded the matrigel membrane in duplicate. The positive control group was taken as 100%. The result was calculated from the difference compared to the positive control group.

### Statistical Analysis

The cell lines were separated in two groups, according to the treatments performed (control and melatonin). First, the results were submitted to analysis of normal distribution using Column statistics and the Gaussian distribution test. The average of densitometric analysis, cell viability and the percentage of total cells with migratory and invasive capacity were subjected to Student's t test. All values were expressed as the average ± Standard Deviation (SD). The value of *P*<0.05 was considered statistically significant. All analyses were performed using GraphPad PRISM5 software (GraphPad Software, Inc., La Jolla, CA).

## Results

### CMT-U229 and MCF-7 cell lines mammospheres

**Fig 1:**
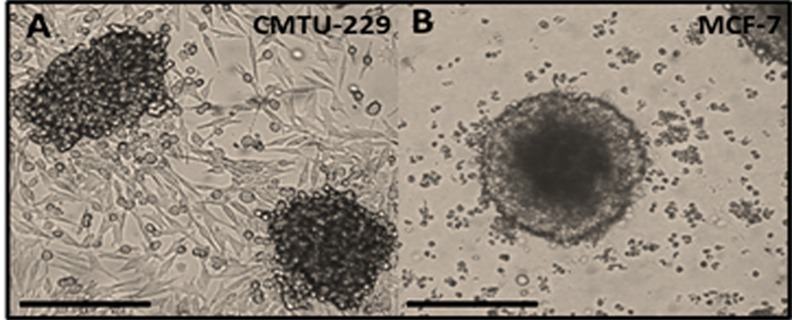
Mammospheres formation in CMT-U229 and MCF-7 cell lines. (**A**) CMT-U229 and (**B**) MCF-7 cell lines in MammoCult^TM^ medium. The original magnification was 40 X. Scale Bar: 10 μm.

The mammospheres were generated from the canine mammary cancer cell line CMT-U229 and human breast cancer cell line MCF-7 in MammoCult^TM^ medium (StemCell Technologies) and efficiently formed compact mammospheres (Fig 1). CMT-U229 and MCF-7 cells were continuously capable of forming mammospheres through repeated subcultures in MammoCult^TM^ medium (StemCell Technologies). The mammosphere formation rate was 0.5.

### Relationship of CMT-U229 and MCF-7 mammospheres and cancer stem cell population

**Fig 2:**
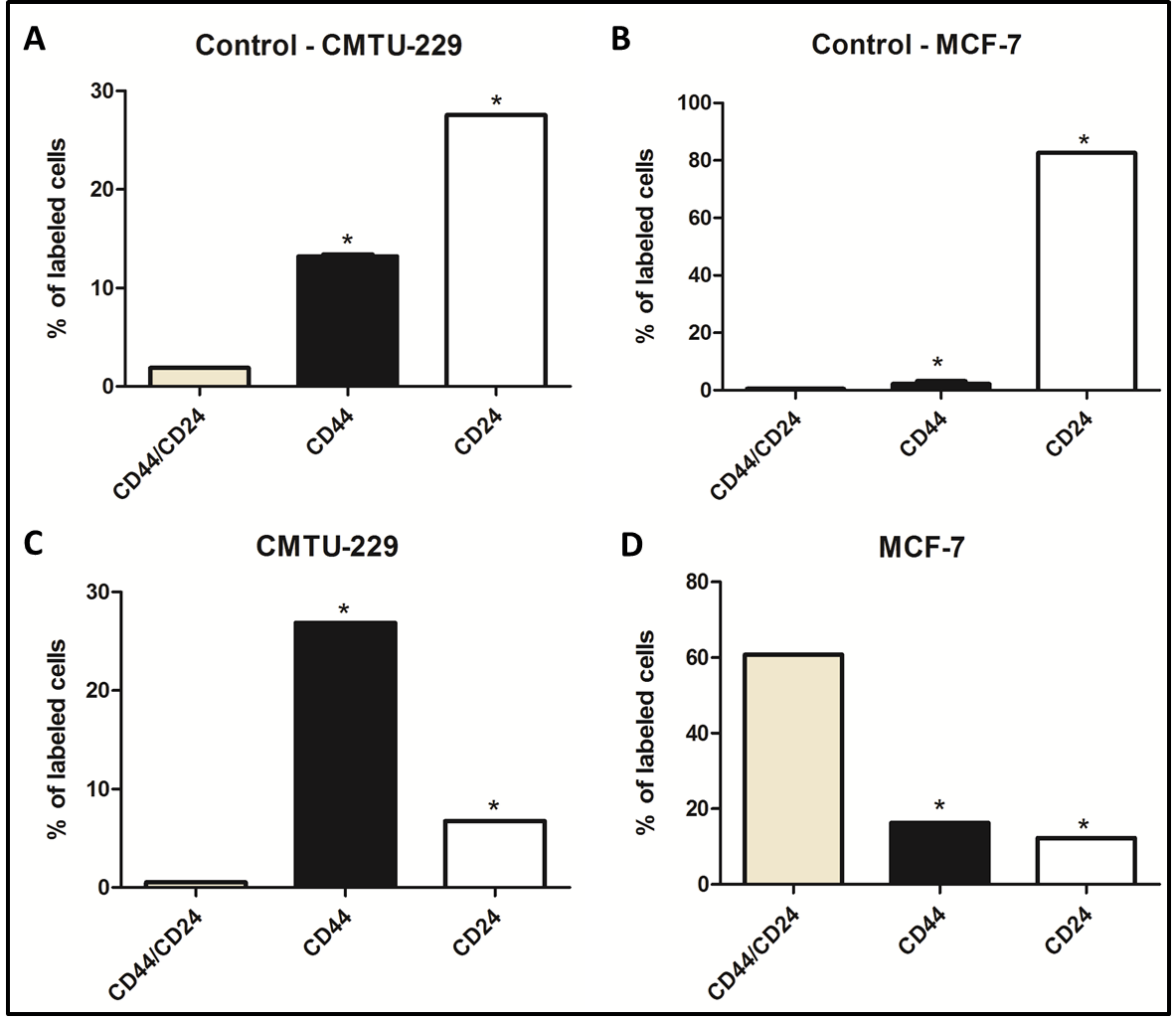
Graphical representation demonstrating the flow cytometry percentage for CD44/CD24 cancer stem cell phenotype in cells. Flow citometry for cells grown in mololayer for control group of (A) CMTU-229 cell line and (B) MCF-7 cell line. Flow citometry for mammospheres for both cell lines (C) CMTU-229 and (D) MCF-7. *P<0.05 Statistical significance compared to CD44 group was determine by Bonferroni.

To confirm the phenotype of breast cancer stem cells (CD44 ^+^ / CD24^- / low^) flow cytometry was performed in mammospheres. For reaction control monolayer of CMTU-229 and MCF-7 cells were used. Our results confirmed the phenotype of breast cancer stem cells. The results indicated that the mammary stem cells population for CMTU-229 cell line was consisted of 26.89% of cell positive for the anti-CD44 antibody and 6.75% for the anti-CD24 antibody (Fig 2C; *P* = 0.0001). Similarly, for the MCF-7 mammospheres 16.29% were positive for CD44 and 12.3% for CD24 (Fig 2D; *P* = 0.0001). However, cells grown in monolayer culture showed the non-stem cell phenotype, in CMTU-229 cell line 13.60% CD44^+^ and 27.59% CD24^-^ (Fig 2A; *P* = 0.0404). The same phenotype was observed for MCF-7 strain, from 0.47% CD44 ^+^ and CD24^-^ 82.69% (Fig 2B; *P* = 0.0001).

### Melatonin reduces cell viability of CMT-U229 and MCF-7 mammospheres

**Fig 3:**
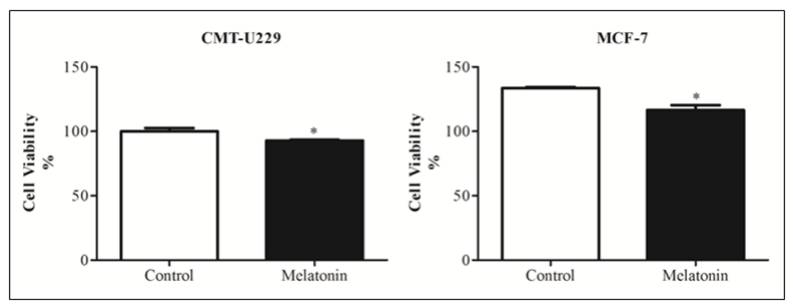
Effect of melatonin on viability of cells from mammospheres. (**A**) CMT-U229 and (**B**) MCF-7 cells were treated with 1 mM of melatonin for 24 h and cell viability was measured by MTT assay. The white column corresponds to control group. Each column represents the mean ± standard error of triplicate experiments. *P<0.05 Statistical significance compared to control group was determine by Student´s t- test.

Thereby, it was performed an MTT assay to estimate the number of viable cells after the treatment with 1 mM melatonin for 24 h. The viability of cells from mammospheres, in both cells lines, was significantly decreased after melatonin treatment when compared to control groups (*P* = 0.0147, *P* = 0.0286, respectively; Fig 3).

### Melatonin induces differential proteins expression in EMT mammospheres process

In order to examine the effects of melatonin on breast cancer stem cells from canine and human cell lines, the protein expression of the cancer stem cell marker OCT4, the epithelial marker E- cadherin, the mesenchymal markers N-cadherin and vimentin, were measured in cells from mammospheres.

**Fig 4:**
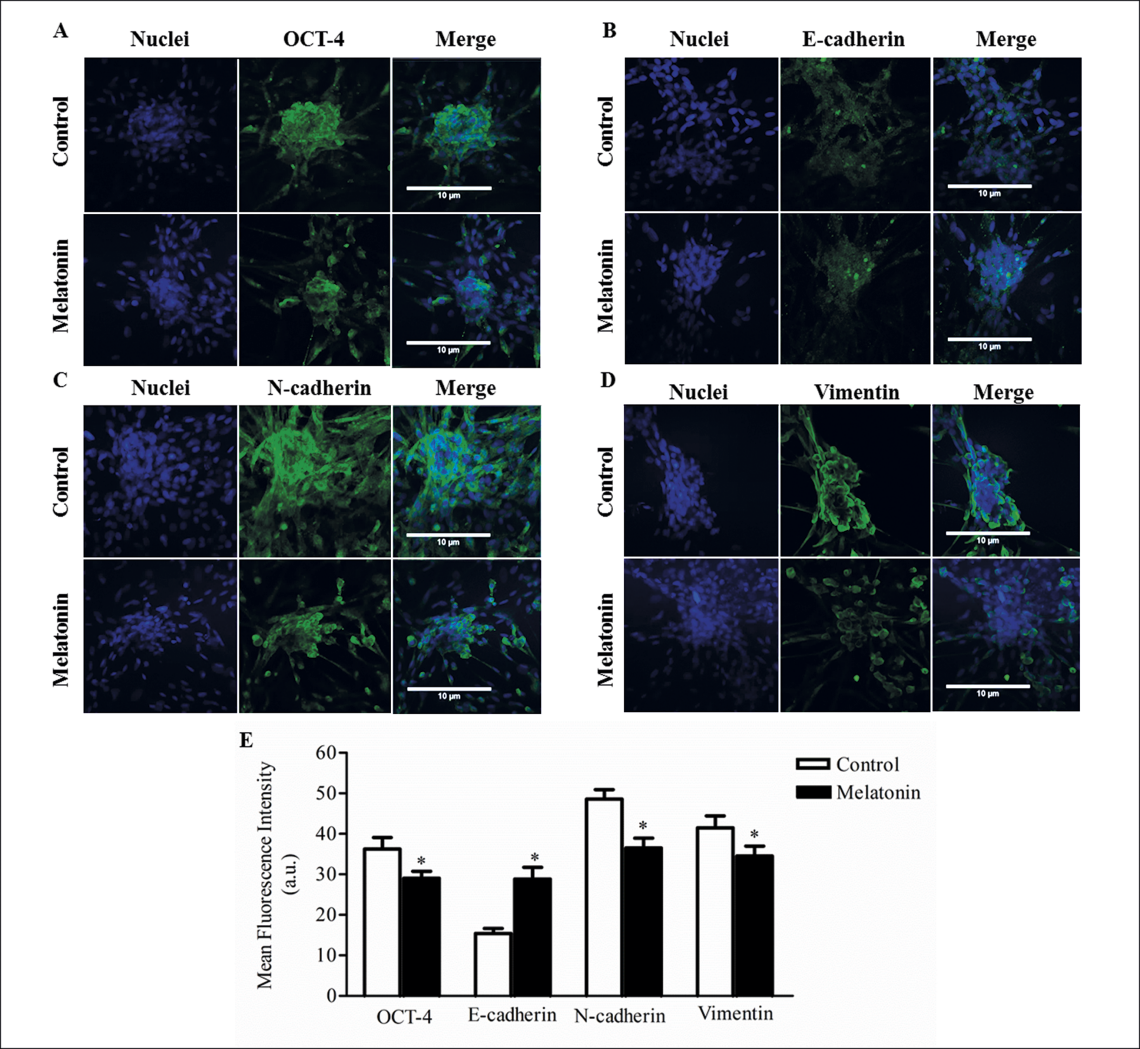
Immunofluorescence. Detection of (**A**) OCT4, (**B**) E-cadherin, (**C**) N-cadherin and (**D**) vimentin in CMT-U229 cells mammospheres after melatonin treatment compared to control groups. **E**. Statistical analysis of OCT4, E-cadherin, N-cadherin and vimentin proteins expression. Data are shown as mean ± standard deviation. The magnification was 40 X. *P<0.05 Statistical significance compared to control group was determine by Student´s t- test.

In CMT-U229 cells treated with 1 mM of melatonin, OCT4 protein expression was significantly decreased compared to the control group (*P* = 0.0426; Fig 4A and 4E) and E-cadherin protein expression increased after melatonin treatment compared to the control group (*P* = 0.0002; Fig 4B and 4E). On the other hand, N-cadherin and vimentin protein expression were significantly decreased in the melatonin treated cells compared to the control groups (*P* = 0.0002; Fig 4C and 4E and *P* = 0.0411; Fig 4D and 4E, respectively).

**Fig 5:**
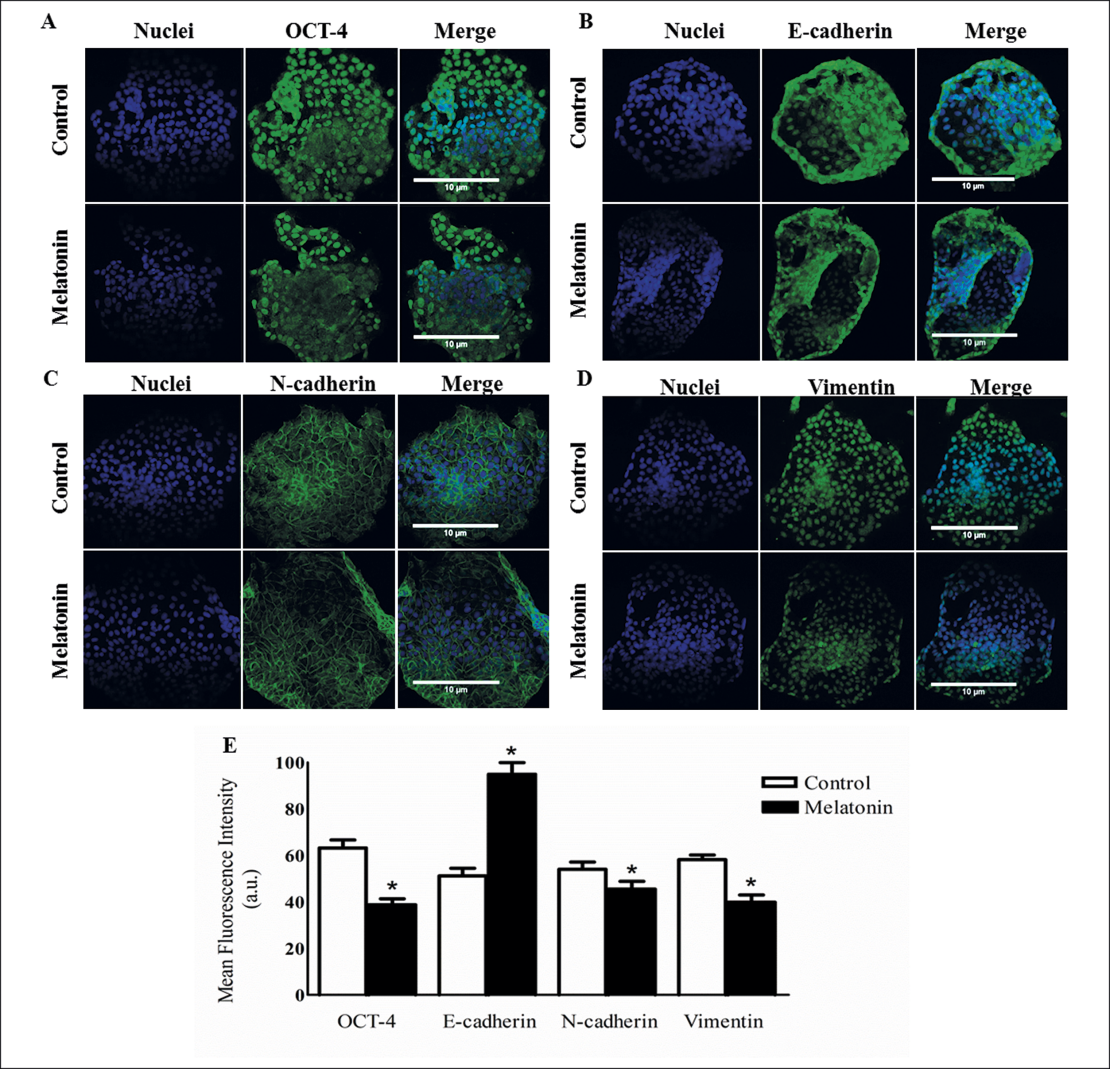
Immunofluorescence. Detection of (**A**) OCT4, (**B**) E-cadherin, (**C**) N-cadherin and (**D**) Vimentin in MCF-7 cells after melatonin treatment compared to control groups. **E**. Statistical analysis of OCT4, E-cadherin, N-cadherin and Vimentin protein expression. Data are shown as mean ± standard deviation. The magnification was 40 X. *P < 0.05 Statistical significance compared to control group was determine by Student´s t- test.

For MCF-7 cells, OCT4 protein expression was also decreased in melatonin treated cells compared to the control group (*P* = 0.0001; Fig 5A and 5E) and E-cadherin protein expression increased after melatonin treatment compared to the control group (*P* = 0.0001; Fig 5B and 5E). For N-cadherin and vimentin proteins, low expression occurred in the treated cells compared to the control groups (*P* = 0.0430; Fig 5C and 5E and *P* = 0.0001; Fig 5D and 5E, respectively).

### Melatonin decreased migration and invasion of CMT-U229 and MCF-7 mammospheres

**Fig 6:**
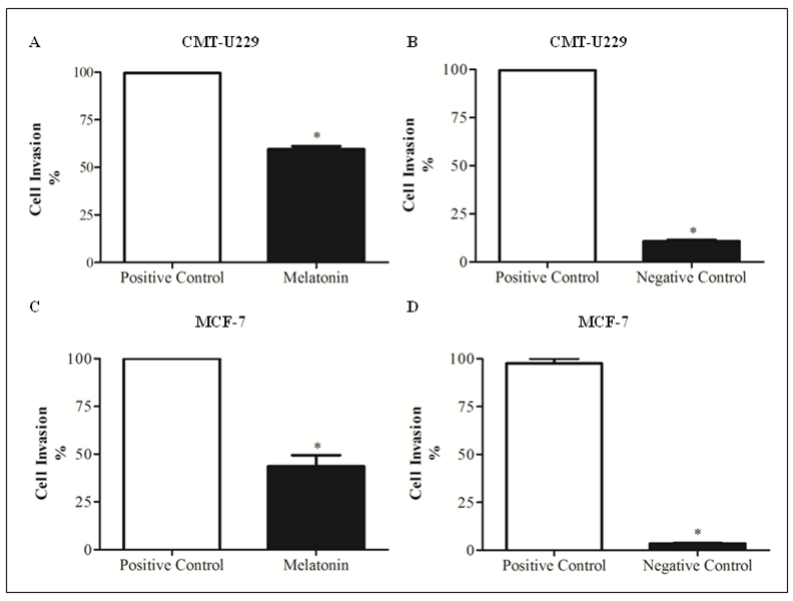
Migration and invasion rate of CMT-U229 and MCF-7 mammospheres after melatonin treatment. (**A**) CMT-U229 and (**C**) MCF-7 cell lines after 24 h of treatment with 1 mM melatonin compared with positive control groups. (**B**) CMT-U229 and (**D**) MCF-7 cell lines correlation between positive and negative control groups. Data are shown as average ± Standard Deviation. *P < 0.05 Statistical significance compared to control group was determine by Student´s t- test.

To confirm the above-mentioned effect of melatonin on canine and human mammospheres, CMT-U229 and MCF-7 cells were treated with 1 mM of melatonin for 24 h and it was verified reduction in migration and invasion in both cell lines (40.4% for CMT-U229 and 56.3% for MCF-7), when compared to the positive control (*P* = 0.0017, *P* = 0.0377, respectively; Fig 6A–6D).

## Discussion

The main purpose of this study was to evaluate the effects of melatonin treatment in breast CSC of canine and human cell lines and the results demonstrated that melatonin has similar activity in the canine CMT-U229 and human MCF-7 cell lines. This fact reinforces the concept that breast tumors in women and female dogs have similar biological characteristics and dogs tumors can be used to better understand the pathogenesis of this disease and valid comparative models for the study of breast tumors [48, 49].

The presence of stem cells in canine [19, 37, 50–54] and human [19,52] breast cancer cell lines have been demonstrated in several studies. CSC exhibit surface markers, whereby the subpopulation of stem cells can be separated from non-stem cells [50]. According to Dontu et al. [12] stemness' of tumor cells is measured *in vitro* by its ability to form mammospheres. As can be seen in this study, canine and human breast cancer efficiently formed mammospheres, consisting of CD44^+^/CD24^low/-^ cells, which confirms the cancer stem cell phenotype. The selection of CSCs from both lineages was performed in serum-free culture medium permissive for growing of stem cells.

Breast cancer cells with CD44^+^/CD24^low/-^ surface phenotype have tumor initiating properties with pluripotency characteristics and invasive capacity. The transition from an epithelial phenotype (CD44^-^/CD24^+^) to a mesenchymal phenotype (CD44^+^/CD24^low/-^) enables the cell to move from the primary tumor to metastatic site [55]. Ponti et al. [10] found that breast cancer cell line that grown as spheroids also had CD44^+^/CD24^low/-^ phenotype and expressed the transcription factor OCT4. OCT4 is a member of the POU family, expressed in embryonic stem cells, germ cells and human stem cells and it is responsible for maintaining an undifferentiated state in the cells [56]. This transcription factor is an essential regulator for self-renewal and pluripotency of embryonic stem cells. Since CSCs share features with embryonic stem cells, it is suggested that transcription factors are expressed commonly in both cells [57].

The expression of OCT4 has an important role in carcinogenesis and provides a possible mechanism by which cancer cells acquire or maintain the therapy resistance phenotype [58]. Linn et al. [58] related overexpression of OCT4 with drug resistance in prostate cancer cell line. Furthermore, overexpression of this gene has also been associated with metastasis and poor prognosis in several types of cancer, including colorectal [59], lung [60] and glioma [61].

In our study, it was showed the expression of OCT4 protein in canine and human breast cancer cell lines, and this expression was decreased after 1 mM of melatonin treatment. Pang et al. [62] and Ferletta et al. [37] also noted OCT4 expression in mammospheres of canine mammary tumor cell lines, REM134 and CMT-U229 avl2, respectively. However, few studies have evaluated the action of melatonin in molecular markers of stem cells [21, 63, 64] working with embryonic stem cells ES-E14TG observed a transient reduction in the expression of OCT4 after treatment with melatonin. The molecular mechanism by which melatonin inhibits the expression of OCT4 is not yet understood.

The CSC have an invasive and metastatic phenotype and can be generated by epithelial mesenchymal transition mechanism (EMT) [17]. Previous studies showed that EMT activation of human neoplastic mammary epithelial cells is associated with enrichment of cells with stem-like properties [55, 62]. Thus, due to the relationship between EMT and cancer stem cell, we verified the presence of EMT molecular markers in CMT-U229 and MCF-7 stem cells. Also, we analyzed the action of melatonin treatment on these EMT markers. Our results showed that the CSCs from both cell lines, CMT-U229 and MCF-7, presented decrease in E-cadherin expression and increase in N-cadherin and vimentin expression. Furthermore, after melatonin treatment, there was an increase of E-cadherin expression and a decrease of N-cadherin and vimentin expression. Some studies have demonstrated low expression of E-cadherin associated with metastasis, lymph nodes involvement and poor prognostic in breast [22, 65] and liver cancer [66]. On the other hand, overexpression of N-cadherin and vimentin is correlated with poor prognosis in several types of cancer, including colorectal [67], bladder [32] and hepatocellular carcinoma [66].

However, few studies have evaluated the action of melatonin on EMT markers. According to our results, Cos et al. [68] found high expression of E-cadherin in MCF-7 cells treated with melatonin. Ma et al. [69] also verified an increase of E-cadherin and survival, in mice with mammary tumor treated with melatonin. According to Mao et al. [33], melatonin has inhibited EMT in MCF-7 cells because it induces the degradation of β-catenin, an E-cadherin repressor, via activation of kinase protein GSK3β. The β-catenin can translocate to the nucleus and complex with the transcription T-cell factor/lymphocyte enhancer factor (TCF/LEF) to induce the transcription of Wnt target genes, including the zinc finger protein Snail, a transcriptional repressor of E-cadherin [70].

Cos et al. [68] found that there was no vimentin expression in both treated and untreated MCF-7 cells after melatonin treatment, probably due to the fact that vimentin is a specific marker of mesenchymal cells. In our study it was observed vimentin expression in both cell lines and its expression decreased after treatment with melatonin. According to Gilles et al. [71] and Mao et al. [33], a signaling model of Wnt/β-catenin also induces the expression of vimentin. Therefore, melatonin may decrease vimentin expression by GSK3β activation and consequential β-catenin degradation. Besides that, melatonin action on N-cadherin expression has been little explored in the literature, but the Wnt/β-catenin signaling model also induces the expression of N-cadherin [72], therefore, melatonin can suppress N-cadherin expression by a similar mechanism to that described above for vimentin.

It was also observed that melatonin decreased the viability of stem cells in both cell lines. Currently, some studies have shown inhibitory effect of melatonin on CSC [34, 35]. Kannen et al. [35] by using colorectal CSCs demonstrated that melatonin reduces cell proliferation and promotes apoptosis of cancer stem cells. Similar results were found by Martin et al. [34], which showed that melatonin induces cell death by autophagy and simultaneously increased the effect of chemotherapy in glioma stem cells.

However, the exact mechanism through which melatonin inhibits cell growth in both *in vitro* and *in vivo* models is not fully understood, although some mechanisms have been proposed. These include induction of apoptosis, changes in the lipidic metabolism and an increase in the activity of natural killer cells, as well as stimulation of cytokines production, such as interleukins IL-2, IL-6, IL-12 and IFN [73].

Some studies also indicate that melatonin is able to reduce migration and invasion in some cancer types, such as glioblastoma [74], hepatocarcinoma [75], lung [76] and breast cancer [68, 77, 78]. According to Cos et al. [68], melatonin treatment reduces the invasiveness of MCF-7 cells, causing a decrease in cell attachment and cell motility, probably by interacting with the estrogen-mediated mechanisms of MCF-7 cells. Ortíz-López et al. [77] verified that melatonin inhibits the migration process and cell invasion in MCF-7 cells via ROCK-1 protein by modulating dynamic cytoskeleton. In turn, Mao et al. [78] found that melatonin exerts an inhibitory effect in breast cancer cell invasion through down-regulation of the p38 pathway, and inhibition of metalloproteinase 2 and 9 expression and activity. According to that, we also found that 1 mM of melatonin reduced the invasiveness of stem cells in both cell lines, CMT-U229 and MCF-7, and this fact may be associated with the anti-metastatic properties of melatonin described above. This result is of great interest since the invasiveness is one of the main characteristics of tumor stem cells.

In conclusion, our results show that the breast cancer stem cells, in both a canine and a human cell line, are responsive to melatonin treatment, reducing the viability and the invasiveness cellular capacity, as well as, the expression of stem cell and EMT markers. Few studies have investigated the melatonin effects on breast cancer stem cells, making this study the first one to simultaneously evaluate canine and human cell lines. In addition, due to the breast CSCs being highly resistant to chemotherapy, drugs that act successfully on this subpopulation can represent an effective therapeutic option for the breast cancer patient.

## Supporting Information

S1 Fig Negative controls of immunofluorescence staining. (JPG)

## References

[1] Jemal A, Bray F, Center MM, Ferlay J, Ward E, Forman D. Global cancer statistics. CA Cancer J Clin. 2011;61(2):69–90. 10.3322/caac.20107 . 21296855

[2] Manuali E, De Giuseppe A, Feliziani F, Forti K, Casciari C, Marchesi MC, et al CA 15–3 cell lines and tissue expression in canine mammary cancer and the correlation between serum levels and tumour histological grade. BMC Vet Res. 2012;8:86 10.1186/1746-6148-8-86 22726603 PMC3412725

[3] Zuccari DAPC, Pavam MV, Terzian ACB, Pereira RS, Ruiz CM, Andrade JC. Immunohistochemical evaluation of e-cadherin, Ki-67 and PCNA in canine mammary neoplasias: Correlation of prognostic factors and clinical outcome. 2008. p. 207–15.

[4] Ma Y, Hao X, Zhang S, Zhang J. The in vitro and in vivo effects of human umbilical cord mesenchymal stem cells on the growth of breast cancer cells. Breast Cancer Res Treat. 2012;133(2):473–85. 10.1007/s10549-011-1774-x . 21947651

[5] Chen K, Huang YH, Chen JL. Understanding and targeting cancer stem cells: therapeutic implications and challenges. Acta Pharmacol Sin. 2013;34(6):732–40. 10.1038/aps.2013.27 23685952 PMC3674516

[6] Luo M, Clouthier SG, Deol Y, Liu S, Nagrath S, Azizi E, et al Breast cancer stem cells: current advances and clinical implications. Methods Mol Biol. 2015;1293:1–49. 10.1007/978-1-4939-2519-3_1 . 26040679

[7] Mitra A, Mishra L, Li S. EMT, CTCs and CSCs in tumor relapse and drug-resistance. Oncotarget. 2015;6(13):10697–711. . 25986923 10.18632/oncotarget.4037 PMC4484413

[8] Bao B, Ahmad A, Azmi AS, Ali S, Sarkar FH. Overview of cancer stem cells (CSCs) and mechanisms of their regulation: implications for cancer therapy. Curr Protoc Pharmacol. 2013;Chapter 14:Unit 14.25. 10.1002/0471141755.ph1425s61 23744710 PMC3733496

[9] Al-Hajj M, Wicha MS, Benito-Hernandez A, Morrison SJ, Clarke MF. Prospective identification of tumorigenic breast cancer cells. Proc Natl Acad Sci U S A. 2003;100(7):3983–8. 10.1073/pnas.0530291100 12629218 PMC153034

[10] Ponti D, Costa A, Zaffaroni N, Pratesi G, Petrangolini G, Coradini D, et al Isolation and in vitro propagation of tumorigenic breast cancer cells with stem/progenitor cell properties. Cancer Res. 2005;65(13):5506–11. 10.1158/0008-5472.CAN-05-0626 . 15994920

[11] Cariati M, Naderi A, Brown JP, Smalley MJ, Pinder SE, Caldas C, et al Alpha-6 integrin is necessary for the tumourigenicity of a stem cell-like subpopulation within the MCF7 breast cancer cell line. Int J Cancer. 2008;122(2):298–304. 10.1002/ijc.23103 . 17935134

[12] Dontu G, Abdallah WM, Foley JM, Jackson KW, Clarke MF, Kawamura MJ, et al In vitro propagation and transcriptional profiling of human mammary stem/progenitor cells. Genes Dev. 2003;17(10):1253–70. 10.1101/gad.1061803 12756227 PMC196056

[13] Jung JW, Park SB, Lee SJ, Seo MS, Trosko JE, Kang KS. Metformin represses self-renewal of the human breast carcinoma stem cells via inhibition of estrogen receptor-mediated OCT4 expression. PLoS One. 2011;6(11):e28068 10.1371/journal.pone.0028068 22132214 PMC3223228

[14] Han XY, Wei B, Fang JF, Zhang S, Zhang FC, Zhang HB, et al Epithelial-mesenchymal transition associates with maintenance of stemness in spheroid-derived stem-like colon cancer cells. PLoS One. 2013;8(9):e73341 10.1371/journal.pone.0073341 24039918 PMC3767831

[15] Grosse-Wilde A, Fouquier d'Hérouël A, McIntosh E, Ertaylan G, Skupin A, Kuestner RE, et al Stemness of the hybrid Epithelial/Mesenchymal State in Breast Cancer and Its Association with Poor Survival. PLoS One. 2015;10(5):e0126522 10.1371/journal.pone.0126522 26020648 PMC4447403

[16] Kalluri R, Weinberg RA. The basics of epithelial-mesenchymal transition. J Clin Invest. 2009;119(6):1420–8. 10.1172/JCI39104 19487818 PMC2689101

[17] Abell AN, Johnson GL. Implications of Mesenchymal Cells in Cancer Stem Cell Populations: Relevance to EMT. Curr Pathobiol Rep. 2014;2(1):21–6. 10.1007/s40139-013-0034-7 25530923 PMC4266994

[18] Hollestelle A, Peeters JK, Smid M, Timmermans M, Verhoog LC, Westenend PJ, et al Loss of E-cadherin is not a necessity for epithelial to mesenchymal transition in human breast cancer. Breast Cancer Res Treat. 2013;138(1):47–57. 10.1007/s10549-013-2415-3 . 23338761

[19] Manuel Iglesias J, Beloqui I, Garcia-Garcia F, Leis O, Vazquez-Martin A, Eguiara A, et al Mammosphere formation in breast carcinoma cell lines depends upon expression of E-cadherin. PLoS One. 2013;8(10):e77281 10.1371/journal.pone.0077281 24124614 PMC3790762

[20] Gheldof A, Berx G. Cadherins and epithelial-to-mesenchymal transition. Prog Mol Biol Transl Sci. 2013;116:317–36. 10.1016/B978-0-12-394311-8.00014-5 . 23481201

[21] Chen A, Beetham H, Black MA, Priya R, Telford BJ, Guest J, et al E-cadherin loss alters cytoskeletal organization and adhesion in non-malignant breast cells but is insufficient to induce an epithelial-mesenchymal transition. BMC Cancer. 2014;14:552 10.1186/1471-2407-14-552 25079037 PMC4131020

[22] Kallergi G, Papadaki MA, Politaki E, Mavroudis D, Georgoulias V, Agelaki S. Epithelial to mesenchymal transition markers expressed in circulating tumour cells of early and metastatic breast cancer patients. Breast Cancer Res. 2011;13(3):R59 10.1186/bcr2896 21663619 PMC3218948

[23] Miyamoto Y, Sakane F, Hashimoto K. N-cadherin-based adherens junction regulates the maintenance, proliferation, and differentiation of neural progenitor cells during development. Cell Adh Migr. 2015;9(3):183–92. 10.1080/19336918.2015.1005466 . 25869655 PMC4594476

[24] Satelli A, Li S. Vimentin in cancer and its potential as a molecular target for cancer therapy. Cell Mol Life Sci. 2011;68(18):3033–46. 10.1007/s00018-011-0735-1 21637948 PMC3162105

[25] Di Bella G, Mascia F, Gualano L, Di Bella L. Melatonin anticancer effects: review. Int J Mol Sci. 2013;14(2):2410–30. 10.3390/ijms14022410 23348932 PMC3587994

[26] Jardim-Perassi BV, Arbab AS, Ferreira LC, Borin TF, Varma NR, Iskander AS, et al Effect of melatonin on tumor growth and angiogenesis in xenograft model of breast cancer. PLoS One. 2014;9(1):e85311 10.1371/journal.pone.0085311 24416386 PMC3887041

[27] Kelleher FC, Rao A, Maguire A. Circadian molecular clocks and cancer. Cancer Lett. 2014;342(1):9–18. 10.1016/j.canlet.2013.09.040 . 24099911

[28] Di Bella G, Mascia F, Colori B. The Di Bella Method (DBM) in the treatment of prostate cancer: a preliminary retrospective study of 16 patients and a review of the literature. Neuro Endocrinol Lett. 2013;34(6):523–8. . 24378460

[29] Lopes JR, Maschio LB, Jardim-Perassi BV, Moschetta MG, Ferreira LC, Martins GR, et al Evaluation of melatonin treatment in primary culture of canine mammary tumors. Oncol Rep. 2015;33(1):311–9. 10.3892/or.2014.3596 . 25384569

[30] Hill SM, Belancio VP, Dauchy RT, Xiang S, Brimer S, Mao L, et al Melatonin: an inhibitor of breast cancer. Endocr Relat Cancer. 2015;22(3):R183–R204. 10.1530/ERC-15-0030 25876649 PMC4457700

[31] Wang Y, Zhou BP. Epithelial-mesenchymal transition in breast cancer progression and metastasis. Chin J Cancer. 2011;30(9):603–11. 10.5732/cjc.011.10226 21880181 PMC3702729

[32] Zhao H, Wu QQ, Cao LF, Qing HY, Zhang C, Chen YH, et al Melatonin inhibits endoplasmic reticulum stress and epithelial-mesenchymal transition during bleomycin-induced pulmonary fibrosis in mice. PLoS One. 2014;9(5):e97266 10.1371/journal.pone.0097266 24818755 PMC4018327

[33] Mao L, Dauchy RT, Blask DE, Slakey LM, Xiang S, Yuan L, et al Circadian gating of epithelial-to-mesenchymal transition in breast cancer cells via melatonin-regulation of GSK3β. Mol Endocrinol. 2012;26(11):1808–20. 10.1210/me.2012-1071 23002080 PMC3487627

[34] Martín V, Sanchez-Sanchez AM, Puente-Moncada N, Gomez-Lobo M, Alvarez-Vega MA, Antolín I, et al Involvement of autophagy in melatonin-induced cytotoxicity in glioma-initiating cells. J Pineal Res. 2014;57(3):308–16. 10.1111/jpi.12170 . 25163989

[35] Kannen V, Marini T, Zanette DL, Frajacomo FT, Silva GE, Silva WA, et al The melatonin action on stromal stem cells within pericryptal area in colon cancer model under constant light. Biochem Biophys Res Commun. 2011;405(4):593–8. 10.1016/j.bbrc.2011.01.074 . 21266165

[36] Zhao J, Dong D, Sun L, Zhang G. Prognostic significance of the epithelial-to-mesenchymal transition markers e-cadherin, vimentin and twist in bladder cancer. Int Braz J Urol. 2014;40(2):179–89. 10.1590/S1677-5538.IBJU.2014.02.07 24856504

[37] Ferletta M, Grawé J, Hellmén E. Canine mammary tumors contain cancer stem-like cells and form spheroids with an embryonic stem cell signature. Int J Dev Biol. 2011;55(7–9):791–9. 10.1387/ijdb.113363mf . 22161835

[38] Qu Y, Han B, Yu Y, Yao W, Bose S, Karlan BY, et al Evaluation of MCF10A as a Reliable Model for Normal Human Mammary Epithelial Cells. PLoS One. 2015;10(7):e0131285 10.1371/journal.pone.0131285 26147507 PMC4493126

[39] Shaw FL, Harrison H, Spence K, Ablett MP, Simões BM, Farnie G, et al A detailed mammosphere assay protocol for the quantification of breast stem cell activity. J Mammary Gland Biol Neoplasia. 2012;17(2):111–7. 10.1007/s10911-012-9255-3 . 22665270

[40] Jardim-Perassi BV, Lourenco MR, Doho GM, Grigolo IH, Gelaleti GB, Ferreira LC, et al Melatonin Regulates Angiogenic Factors Under Hypoxia in Breast Cancer Cell Lines. Anticancer Agents Med Chem. 2015 . 25963143 10.2174/1871520615666150511094201

[41] Borin TF, Arbab AS, Gelaleti GB, Ferreira LC, Moschetta MG, Jardim-Perassi BV, et al Melatonin decreases breast cancer metastasis by modulating ROCK-1 expression. J Pineal Res. 2015 10.1111/jpi.12270 . 26292662 PMC4996347

[42] Dai M, Cui P, Yu M, Han J, Li H, Xiu R. Melatonin modulates the expression of VEGF and HIF-1 alpha induced by CoCl2 in cultured cancer cells. J Pineal Res. 2008;44(2):121–6. 10.1111/j.1600-079X.2007.00498.x . 18289162

[43] Park SY, Jang WJ, Yi EY, Jang JY, Jung Y, Jeong JW, et al Melatonin suppresses tumor angiogenesis by inhibiting HIF-1alpha stabilization under hypoxia. J Pineal Res. 2010;48(2):178–84. . 20449875 10.1111/j.1600-079x.2009.00742.x

[44] Alvarez-García V, González A, Alonso-González C, Martínez-Campa C, Cos S. Melatonin interferes in the desmoplastic reaction in breast cancer by regulating cytokine production. J Pineal Res. 2012;52(3):282–90. 10.1111/j.1600-079X.2011.00940.x . 22151118

[45] Carbajo-Pescador S, Ordoñez R, Benet M, Jover R, García-Palomo A, Mauriz JL, et al Inhibition of VEGF expression through blockade of Hif1α and STAT3 signalling mediates the anti-angiogenic effect of melatonin in HepG2 liver cancer cells. Br J Cancer. 2013;109(1):83–91. 10.1038/bjc.2013.285 23756865 PMC3708553

[46] Cui P, Yu M, Peng X, Dong L, Yang Z. Melatonin prevents human pancreatic carcinoma cell PANC-1-induced human umbilical vein endothelial cell proliferation and migration by inhibiting vascular endothelial growth factor expression. J Pineal Res. 2012;52(2):236–43. 10.1111/j.1600-079X.2011.00933.x . 21913973

[47] González A, Alvarez-García V, Martínez-Campa C, Alonso-González C, Cos S. Melatonin promotes differentiation of 3T3-L1 fibroblasts. J Pineal Res. 2012;52(1):12–20. 10.1111/j.1600-079X.2011.00911.x . 21718362

[48] Uva P, Aurisicchio L, Watters J, Loboda A, Kulkarni A, Castle J, et al Comparative expression pathway analysis of human and canine mammary tumors. BMC Genomics. 2009;10:135 10.1186/1471-2164-10-135 19327144 PMC2670324

[49] Barbieri F, Thellung S, Ratto A, Carra E, Marini V, Fucile C, et al In vitro and in vivo antiproliferative activity of metformin on stem-like cells isolated from spontaneous canine mammary carcinomas: translational implications for human tumors. BMC Cancer. 2015;15:228 10.1186/s12885-015-1235-8 25884842 PMC4397725

[50] Torres CG, Olivares A, Stoore C. Simvastatin exhibits antiproliferative effects on spheres derived from canine mammary carcinoma cells. Oncol Rep. 2015;33(5):2235–44. 10.3892/or.2015.3850 . 25778435

[51] Magalhães GM, Terra EM, de Oliveira Vasconcelos R, de Barros Bandarra M, Moreira PR, Rosolem MC, et al Immunodetection of cells with a CD44+/CD24- phenotype in canine mammary neoplasms. BMC Vet Res. 2013;9:205 10.1186/1746-6148-9-205 24119896 PMC3852738

[52] Owens TW, Naylor MJ. Breast cancer stem cells. Front Physiol. 2013;4:225 10.3389/fphys.2013.00225 23986719 PMC3753536

[53] Cocola C, Anastasi P, Astigiano S, Piscitelli E, Pelucchi P, Vilardo L, et al Isolation of canine mammary cells with stem cell properties and tumour-initiating potential. Reprod Domest Anim. 2009;44 Suppl 2:214–7. 10.1111/j.1439-0531.2009.01413.x . 19754572

[54] Michishita M, Akiyoshi R, Suemizu H, Nakagawa T, Sasaki N, Takemitsu H, et al Aldehyde dehydrogenase activity in cancer stem cells from canine mammary carcinoma cell lines. Vet J. 2012;193(2):508–13. 10.1016/j.tvjl.2012.01.006 . 22326935

[55] Mani SA, Guo W, Liao MJ, Eaton EN, Ayyanan A, Zhou AY, et al The epithelial-mesenchymal transition generates cells with properties of stem cells. Cell. 2008;133(4):704–15. 10.1016/j.cell.2008.03.027 18485877 PMC2728032

[56] Gerrard L, Zhao D, Clark AJ, Cui W. Stably transfected human embryonic stem cell clones express OCT4-specific green fluorescent protein and maintain self-renewal and pluripotency. Stem Cells. 2005;23(1):124–33. 10.1634/stemcells.2004-0102 . 15625129

[57] Dick JE. Stem cell concepts renew cancer research. Blood. 2008;112(13):4793–807. 10.1182/blood-2008-08-077941 . 19064739

[58] Linn DE, Yang X, Sun F, Xie Y, Chen H, Jiang R, et al A Role for OCT4 in Tumor Initiation of Drug-Resistant Prostate Cancer Cells. Genes Cancer. 2010;1(9):908–16. 10.1177/1947601910388271 21779471 PMC3092260

[59] Saigusa S, Tanaka K, Toiyama Y, Yokoe T, Okugawa Y, Ioue Y, et al Correlation of CD133, OCT4, and SOX2 in rectal cancer and their association with distant recurrence after chemoradiotherapy. Ann Surg Oncol. 2009;16(12):3488–98. 10.1245/s10434-009-0617-z . 19657699

[60] Chiou SH, Wang ML, Chou YT, Chen CJ, Hong CF, Hsieh WJ, et al Coexpression of Oct4 and Nanog enhances malignancy in lung adenocarcinoma by inducing cancer stem cell-like properties and epithelial-mesenchymal transdifferentiation. Cancer Res. 2010;70(24):10433–44. 10.1158/0008-5472.CAN-10-2638 . 21159654

[61] Guo Y, Liu S, Wang P, Zhao S, Wang F, Bing L, et al Expression profile of embryonic stem cell-associated genes Oct4, Sox2 and Nanog in human gliomas. Histopathology. 2011;59(4):763–75. 10.1111/j.1365-2559.2011.03993.x . 22014056

[62] Pang LY, Cervantes-Arias A, Else RW, Argyle DJ. Canine Mammary Cancer Stem Cells are Radio- and Chemo- Resistant and Exhibit an Epithelial-Mesenchymal Transition Phenotype. Cancers (Basel). 2011;3(2):1744–62. 10.3390/cancers3021744 24212780 PMC3757388

[63] Yoo YM, Jung EM, Choi KC, Jeung EB. Effect of melatonin on mRNA expressions of transcription factors in murine embryonic stem cells. Brain Res. 2011;1385:1–7. 10.1016/j.brainres.2011.02.047 . 21349252

[64] Gao S, Wang ZL, Di KQ, Chang G, Tao L, An L, et al Melatonin improves the reprogramming efficiency of murine-induced pluripotent stem cells using a secondary inducible system. J Pineal Res. 2013;55(1):31–9. 10.1111/jpi.12047 . 23506542

[65] Yu Z, Sun M, Jin F, Xiao Q, He M, Wu H, et al Combined expression of ezrin and E-cadherin is associated with lymph node metastasis and poor prognosis in breast cancer. Oncol Rep. 2015;34(1):165–74. 10.3892/or.2015.3967 . 25955302

[66] Zhai X, Zhu H, Wang W, Zhang S, Zhang Y, Mao G. Abnormal expression of EMT-related proteins, S100A4, vimentin and E-cadherin, is correlated with clinicopathological features and prognosis in HCC. Med Oncol. 2014;31(6):970 10.1007/s12032-014-0970-z . 24781336

[67] Yan X, Yan L, Liu S, Shan Z, Tian Y, Jin Z. N-cadherin, a novel prognostic biomarker, drives malignant progression of colorectal cancer. Mol Med Rep. 2015;12(2):2999–3006. 10.3892/mmr.2015.3687 . 25936636

[68] Cos S, Fernández R, Güézmes A, Sánchez-Barceló EJ. Influence of melatonin on invasive and metastatic properties of MCF-7 human breast cancer cells. Cancer Res. 1998;58(19):4383–90. . 9766668

[69] Ma C, Li LX, Zhang Y, Xiang C, Ma T, Ma ZQ, et al Protective and sensitive effects of melatonin combined with adriamycin on ER+ (estrogen receptor) breast cancer. Eur J Gynaecol Oncol. 2015;36(2):197–202. . 26050360

[70] Zhou BP, Deng J, Xia W, Xu J, Li YM, Gunduz M, et al Dual regulation of Snail by GSK-3beta-mediated phosphorylation in control of epithelial-mesenchymal transition. Nat Cell Biol. 2004;6(10):931–40. 10.1038/ncb1173 . 15448698

[71] Gilles C, Polette M, Mestdagt M, Nawrocki-Raby B, Ruggeri P, Birembaut P, et al Transactivation of vimentin by beta-catenin in human breast cancer cells. Cancer Res. 2003;63(10):2658–64. . 12750294

[72] Howard S, Deroo T, Fujita Y, Itasaki N. A positive role of cadherin in Wnt/β-catenin signalling during epithelial-mesenchymal transition. PLoS One. 2011;6(8):e23899 10.1371/journal.pone.0023899 21909376 PMC3166074

[73] Sainz RM, Mayo JC, Rodriguez C, Tan DX, Lopez-Burillo S, Reiter RJ. Melatonin and cell death: differential actions on apoptosis in normal and cancer cells. Cell Mol Life Sci. 2003;60(7):1407–26. 10.1007/s00018-003-2319-1 . 12943228 PMC11138606

[74] Zhang Y, Liu Q, Wang F, Ling EA, Liu S, Wang L, et al Melatonin antagonizes hypoxia-mediated glioblastoma cell migration and invasion via inhibition of HIF-1α. J Pineal Res. 2013;55(2):121–30. 10.1111/jpi.12052 . 23551342

[75] Ordoñez R, Carbajo-Pescador S, Prieto-Dominguez N, García-Palomo A, González-Gallego J, Mauriz JL. Inhibition of matrix metalloproteinase-9 and nuclear factor kappa B contribute to melatonin prevention of motility and invasiveness in HepG2 liver cancer cells. J Pineal Res. 2014;56(1):20–30. 10.1111/jpi.12092 . 24117795

[76] Zhou Q, Gui S, Wang Y. Melatonin inhibits the migration of human lung adenocarcinoma A549 cell lines involving JNK/MAPK pathway. PLoS One. 2014;9(7):e101132 10.1371/journal.pone.0101132 24992189 PMC4084631

[77] Ortíz-López L, Morales-Mulia S, Ramírez-Rodríguez G, Benítez-King G. ROCK-regulated cytoskeletal dynamics participate in the inhibitory effect of melatonin on cancer cell migration. J Pineal Res. 2009;46(1):15–21. 10.1111/j.1600-079X.2008.00600.x . 18482340

[78] Mao L, Yuan L, Slakey LM, Jones FE, Burow ME, Hill SM. Inhibition of breast cancer cell invasion by melatonin is mediated through regulation of the p38 mitogen-activated protein kinase signaling pathway. Breast Cancer Res. 2010;12(6):R107 10.1186/bcr2794 21167057 PMC3046452

