# Supplementary figures and images for: Effect of Melatonin in Epithelial Mesenchymal Transition Markers and Invasive Properties of Breast Cancer Stem Cells of Canine and Human Cell Lines
Source: PLoS One. 2016 Mar 2;11(3):e0150407. doi: 10.1371/journal.pone.0150407 (PMC4774906; doi:10.1371/journal.pone.0150407)

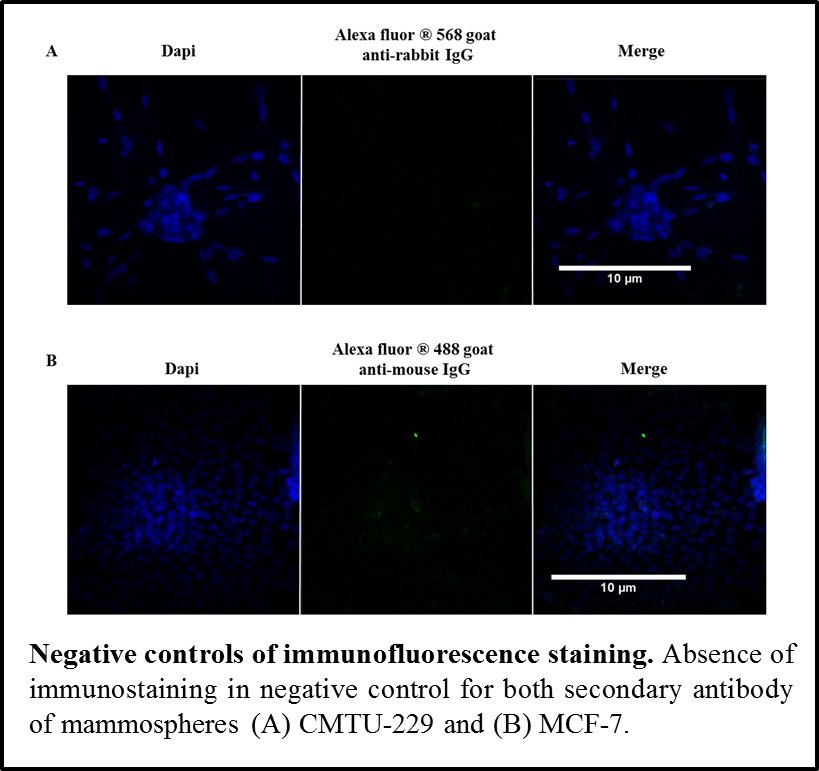

Supplement: S1 Fig — (JPG) [file pone.0150407.s001.jpg]
